# Associations Between Binocular Depth Perception and Performance Gains in Laparoscopic Skill Acquisition

**DOI:** 10.3389/fnhum.2021.675700

**Published:** 2021-10-05

**Authors:** Adamantini Hatzipanayioti, Sebastian Bodenstedt, Felix von Bechtolsheim, Isabel Funke, Florian Oehme, Marius Distler, Jürgen Weitz, Stefanie Speidel, Shu-Chen Li

**Affiliations:** ^1^Centre for Tactile Internet With Human-in-the-Loop, Technische Universität Dresden, Dresden, Germany; ^2^Lifespan Developmental Neuroscience, Faculty of Psychology, Technische Universität Dresden, Dresden, Germany; ^3^Division of Translational Surgical Oncology, National Center for Tumor Diseases Partner Site Dresden, Dresden, Germany; ^4^Department of Visceral, Thoracic and Vascular Surgery, University Hospital Carl Gustav Carus, Technische Universität Dresden, Dresden, Germany

**Keywords:** depth perception, random-dot stereograms, laparoscopic training, skill acquisition, psychometric curve

## Abstract

The ability to perceive differences in depth is important in many daily life situations. It is also of relevance in laparoscopic surgical procedures that require the extrapolation of three-dimensional visual information from two-dimensional planar images. Besides visual-motor coordination, laparoscopic skills and binocular depth perception are demanding visual tasks for which learning is important. This study explored potential relations between binocular depth perception and individual variations in performance gains during laparoscopic skill acquisition in medical students naïve of such procedures. Individual differences in perceptual learning of binocular depth discrimination when performing a random dot stereogram (RDS) task were measured as variations in the slope changes of the logistic disparity psychometric curves from the first to the last blocks of the experiment. The results showed that not only did the individuals differ in their depth discrimination; the extent with which this performance changed across blocks also differed substantially between individuals. Of note, individual differences in perceptual learning of depth discrimination are associated with performance gains from laparoscopic skill training, both with respect to movement speed and an efficiency score that considered both speed and precision. These results indicate that learning-related benefits for enhancing demanding visual processes are, in part, shared between these two tasks. Future studies that include a broader selection of task-varying monocular and binocular cues as well as visual-motor coordination are needed to further investigate potential mechanistic relations between depth perceptual learning and laparoscopic skill acquisition. A deeper understanding of these mechanisms would be important for applied research that aims at designing behavioral interventions for enhancing technology-assisted laparoscopic skills.

## Introduction

Depth perception is important for us to behave and act in the three-dimensional (3D) environment surrounding us. This ability plays an important role in many aspects of our everyday life. First and foremost, it allows us to physically navigate in 3D environments without bumping into obstacles and helps us to judge the distances, speeds, and sizes of objects around us to interact precisely with them. Moreover, intact stereoscopic vision is a special prerequisite for certain professions. For example, drivers (Bauer et al., 2001), fire-fighters (Sheedy, 1984), and pilots (DeHaan, 1982; DeLucia and Task, 1995) need to be able to judge relative distances and perceive object locations in space precisely (Sheedy, 1993). Yet the ability to perceive depth can differ substantially between individuals (Westerman and Cribbin, 1998). The visual system integrates a number of cues to estimate depth. There are two categories of information for detecting depth, cues that are available from the input of one eye (monocular) and cues that require inputs from both eyes (binocular). Monocular cues consist of static information, including relative size, perspective, interposition, lighting, and focus as well as dynamic information such as motion parallax. Binocular cues include disparity and vergence (Howard and Rogers, 2012; Iehisa et al., 2020). In this study, we explored the potential relation between individual differences in the effects of learning on binocular disparity discrimination and laparoscopic skills.

Our ability to make use of subtle differences between visual images received by our left and right eyes for perceiving stereoscopic depth is crucial to the visual perception of 3D space (Parker, 2007). Binocular depth perception relies on the process of stereopsis, which gauges the depth point within an image by perceiving angular disparity between stimulus-induced visual images that are registered on the left and the right retina. Here, we concentrated on the role of binocular depth perception in laparoscopic surgery, which is one of the minimally invasive procedures that are commonly used for a variety of surgical treatments (Simillis et al., 2019). Laparoscopic surgery involves difficult psychomotor activities, and a very substantial amount of first-hand experiences is necessary to obtain proficiency. Take laparoscopic rectal cancer surgeries as an example, about 60 to 80 operations are needed for gaining the expertise (Son et al., 2010). To ensure high-quality patient care, it is crucial to understand factors that may affect individual differences in learning such skills for effective and efficient training (Vedula et al., 2017). To this end, we investigated whether individual differences in the effects of learning on binocular depth discrimination may be associated with between-person variations of performance gains in laparoscopic surgical skill acquisition. Discerning this association as a starting inquiry could motivate further basic research on potential mechanistic relations between depth perceptual learning and laparoscopic skill acquisition, which may have implications for applied research on designing perceptual training paradigms for improving surgical education and for developing visually augmented technologies for laparoscopic surgeries.

Laparoscopic surgery has several advantages over conventional open surgery, such as less damage to healthy tissues, reduced pain, and lower risk of infection. A recent meta-analytic study that compared 29 randomized control trials of rectal cancer resection reported that, although conventional open and laparoscopic procedures yield comparative perioperative morbidity and long-term survival, laparoscopic surgery results in shorter hospital stays and may improve postoperative recovery (Simillis et al., 2019). Notwithstanding these patient benefits, laparoscopic surgeries pose additional visual and sensorimotor challenges to the surgeons due to the restricted two-dimensional (2D) field of view, the inverse motion of laparoscopic instruments, and reduction of haptic feedback. Focusing here on visual information processing, a key challenge in laparoscopic procedure relative to traditional open surgery is the demand of adjusting to the two-dimensional (2D) view of the operative field. In natural viewing situations, depth perception allows us to make judgments about the position of objects from one another and from our body. In laparoscopic surgery, however, when using a single-lens laparoscope, the 3D anatomy of the organs of the patient is shown as planar images on 2D monitors. Such images remove 3D depth cues, resulting in a lack of binocular information while maintaining only limited monoscopic visual cues, such as relative size, occlusion, interposition, shading and lighting, textual gradients, and motion parallax, on which surgeons rely on to infer depth in the operating field (Su et al., 2016; Cahais et al., 2017). These monoscopic visual cues are further degraded by the monitors. Therefore, they are not easily available to the surgeons. Moreover, compared to natural viewing, the limited field of view in laparoscopic surgery restricts the surgeons from seeing the whole operating field, which could lead to objects appearing closer than they are. Additionally, the stationary images from the 2D monitors significantly reduce motion depth cues, such as motion parallax, which normally, would allow the surgeon to dynamically explore the scene from many vantage points. Another disadvantage of the 2D monitors which in turn, contributes to the reduction of one important depth cue comes from the relationship between the position of the lens and the laparoscope. Although using both systems makes the illumination of internal organs possible, it also results in a shadowless operative field (Bogdanova et al., 2016). These limitations pose further difficulties for precise eye-hand coordination and for perceiving motion in depth. Working under such restrictions, the task of the surgeon in inferring depth information from 2D display during laparoscopic surgeries is very challenging. Misjudging depth and reduced haptic feedback could lead to target overshooting in laparoscopic procedures, which can have serious impacts on patient safety and health (Breedveld et al., 1999; Bogdanova et al., 2016). Furthermore, reduced depth cues in laparoscopy also result in an increased procedural time for medical students and novice surgeons (Perkins et al., 2002; de Almeida et al., 2018). Thus, a better understanding of potential relations between individual differences in the effects of learning on binocular depth perception and laparoscopic skills might help improve training programs for such skills.

According to the recent consensus of the European Association of Endoscopic Surgery (Arezzo et al., 2019), the problem of poor depth cues in the 2D images can, in part, be improved by advanced technological supports (e.g., with double lenses or chips) to render 3D laparoscopic images. In an early study, van Bergen et al. (1998) compared surgeons trained in five standardized laparoscopic tasks using both 2D and 3D visual displays and showed that surgeons made less errors and were faster in performing the tasks when a 3D display was used. Similarly, Bhayani and Andriole (2005) demonstrated that novice surgeons showed a higher preference for 3D over 2D visualization systems. However, it should be underscored that, although 3D-displays have been developed, not all current operating theaters have access to 3D visualization systems to support surgical tasks. Thus, laparoscopic surgeons must have surgical proficiency with different types of display modes. Furthermore, currently, the benefit of 3D-display is still debated (see review in Beattie et al., 2020). For instance, recent empirical results have shown that, while 3D-display reduces speed and error during the training phase, these benefits do not transfer to the testing situation with a related but not-yet-learned task after training under either 2D- or 3D-display mode (Beattie et al., 2020). The benefit of 3D display on learning during training also does not contribute to enhanced laparoscopic skill in general; instead, training with 2D-display yielded more effective skill transfer when shifting to the 3D-display mode, since under the 2D condition, users learned to use both primary and secondary depth cues (Poudel et al., 2017; Harada et al., 2018; Beattie et al., 2020).

Independent of display modes, reconstructing 3D anatomy of the organs of the patient, perceiving motion trajectories of the instruments (laparoscopic graspers) from 2D laparoscopic images or processing 3D-rendered visual displays would rely on the ability of binocular depth perception. Thus, further research on individual differences of depth perception, and its relation to performance outcomes of laparoscopic skill acquisition would be informative for applied research on medical education and technologies. Of particular relevance here is the evidence from studies of perceptual learning, which indicates that binocular vision can still be improved even in adulthood (see Başgöze et al., 2018 for review). Previous findings from human and animal studies of perceptual learning, in general, have also revealed that repeated exposure to similar types of sensory stimuli may enhance perceptual discrimination through the recruitment of brain regions relevant to higher-order perceptual decision (Dosher and Lu, 1999; Skrandies and Jedynak, 1999; Gilbert et al., 2001; Fahle, 2005). In the case of depth perception, an early psychophysical study indicated that stereoscopic depth perception can be modified only after a short period of training (Wallach and Karsh, 1963). Evidence from a more recent animal study (Chowdhury and DeAngelis, 2008) further supports this effect in primates and revealed that relative depth discrimination learning could result in redistributions of the involved visual and cognitive brain regions during depth perception. Specifically, after performing a relative depth discrimination task across several experimental blocks, depth perception becomes less dependent on sensory sensitivity of the extrastriate visual cortex (also known as area MT), while the contribution of parietal and frontal brain regions involved in perceptual decisions increases. Given these findings and the fact that the precision of detecting depth (i.e., stereoacuity) can differ substantially between persons (Westerman and Cribbin, 1998), a question that arises is whether individual differences in the effects of learning on depth discrimination may be associated with variations in training outcomes of laparoscopic skill acquisition.

Previous studies on the relation between visual perception and laparoscopic skills have mainly focused on individual traits (i.e., baseline abilities instead of the potential for learning-related improvement) and relied mostly on paper-based tests of either general visual-spatial aptitude (e.g., Buckley et al., 2014) or stereoacuity (e.g., Sakata et al., 2017a,b) that was usually assessed with the Randot^®^Stereotest or the Titmus Fly^®^ Stereotest (STEREO OPTICAL). Thus far, findings from studies using these tests are inconsistent. Another way to assess individual differences in binocular vision is to use computer-generated stimuli. In this context, the Random Dot Stereograms (RDS) viewed through a stereoscope have been known since the early 20th century and are used in laboratory settings to study depth perception (Julesz, 1971). In particular, the RDS task is broadly used by researchers of vision because the stereograms isolate binocular disparity (i.e., the positional difference between images deriving from both eyes) by eliminating monocular cues (e.g., texture, shading, etc.). Humans are sensitive to detecting binocular disparities with an average of only a few seconds of arc (Howard, 1919). Evidence from neurobiological studies has revealed the existence of binocular neurons, which are sensitive to stereoscopic stimulations (Ohzawa et al., 1990; Fleet et al., 1996; Parker, 2007). In clinical populations, research findings showed that visual-evoked brain potentials elicited by computer-generated dynamic RDS were reduced and delayed in people with stereovision deficiencies, such as amblyopia and strabismus (Skrandies and Vomberg, 1985; Skrandies, 2009). Significant individual differences have also been found in healthy individuals by using the anti-correlated RDS task, whereas some individuals could perceive reversed depth, while others could not (Read and Eagle, 2000). To the best of our knowledge, prior studies that applied different variants of the RDS task to study depth perception have not investigated the potential link between individual differences in the effects of learning on depth discrimination and variations in performance outcomes from laparoscopic skill acquisition.

### Study Overview

This study aimed to investigate whether individual differences in perceptual learning of binocular depth discrimination may be associated with variations of performance gains resulted from laparoscopic skill training. We programmed a depth discrimination task with correlated RDS generated by the computer. This task allowed us to assess individual differences in depth discrimination and how this may improve after five blocks of repeated exposure to RDS stimuli. Previous studies have established evidence for depth discrimination learning in the RDS paradigm. Several variants of the RDS tasks (e.g., correlated, anti-correlated, or mixed-correlated) have been established as common paradigms for studying different aspects of depth perception (e.g., O'Toole and Kersten, 1992; Gantz et al., 2007; Henriksen et al., 2016; Asher and Hibbard, 2018; Li and Ackermann, 2018). For instance, the time to perceive depth (response latency) reduces with a few blocks of repeated exposure to the stimuli (O'Toole and Kersten, 1992), and the stereo threshold decreases with extensive practice across thousands of trials (Gantz et al., 2007). In this study, we designed our task (see details in the method section) to be similar to one variant of the RDS tasks used in a recent study on mechanisms of depth perception by Asher and Hibbard (2018). As a screening test and for the purpose of comparison, we also measured individual differences in stereoacuity by using a Stereotest (the Titmus Fly^®^ Stereotest, STEREO OPTICAL) that is commonly used for diagnosing stereopsis in clinical settings (e.g., Fricke and Siderov, 1997; Fawcett and Birch, 2003). Such tests have also been used to discern potential relations between individual differences in stereoacuity and laparoscopic skills (e.g., Sakata et al., 2017a,b). Furthermore, the participants of this study also underwent four sessions of laparoscopic skill training, following the curriculum of *Fundamentals of Laparoscopic Surgery* (Peters et al., 2004). In light of earlier evidence showing that perceptual learning of depth discrimination may also reflect influences of perceptual attention (Sowden et al., 1996) or perceptual decision mechanisms that are subserved by higher-order cognitive brain regions (e.g., the parietal cortex) on depth perception (Chowdhury and DeAngelis, 2008), we expected that individual differences in depth discrimination learning will be associated with variations in performance gains of laparoscopic skill acquisition. Besides basic sensory processes, other factors, such as higher-order perceptual decision processes, may also contribute to this association.

## Methods and Materials

### Participants

Twenty-one medical students (16 females, 5 males), with a mean age of 24.05 years (SD = 2.85), from the Faculty of Medicine Carl Gustav Carus at Technische Universität Dresden participated in the study as a part of their enrollment in one of the courses on laparoscopic surgical skills. The female-to-male ratio in our sample in part reflects the distributions of medical student populations (about 62% females and 38% males) in Germany and at our university (see data from Federal Statistics Office in Germany, https://www.destatis.de/DE/Themen/Gesellschaft-Umwelt/Bildung-Forschung-Kultur/Hochschulen/Tabellen/lrbil05.html). Informed consent was obtained from each of the participants prior to study participation. All the participants reported normal or corrected to normal vision, and none of them reported having any medical history of eye diseases, with the exception of one female participant who reported absence of stereo vision due to a past eye injury. Since we were interested in individual differences in binocular disparity discrimination, this participant did not take part in further experimental procedures. Before the experiment began, the participants were screened for the presence of stereopsis using the graded-circle test of the Titmus Fly^®^ Stereotest (STEREO OPTICAL), which can assess stereopsis in values ranging 400–20 arc sec (from gross to fine acuity) according to the test manual (Deepa et al., 2019). The measured stereoacuity level in our sample reflected this distribution. The average of assessed stereoacuity in our sample across all the participants was 40.1 arc sec (SD = 36.1, range = 20–160). Although three participants included in our sample showed reduced stereoacuity (i.e., had thresholds < 40 arc sec, with their values equal to 50, 100, and 160, respectively), their performances in the main tasks examined in the study (the RDS task and the laparoscopic skill task) were comparable to other participants in the sample (i.e., within 2.5 SD of the group means). A recent population-based study of over 200 college students showed that only 13% of the student population met the stereoacuity level of 20 arc sec or below, 44% of the students were at the level between 25 and 40 arc sec, and 43% were at the level of 50 arc sec and above (Deepa et al., 2019). The measured stereoacuity level in our sample reflected this distribution.

### Depth Discrimination Task With Correlated RDS

**Figure 1:**
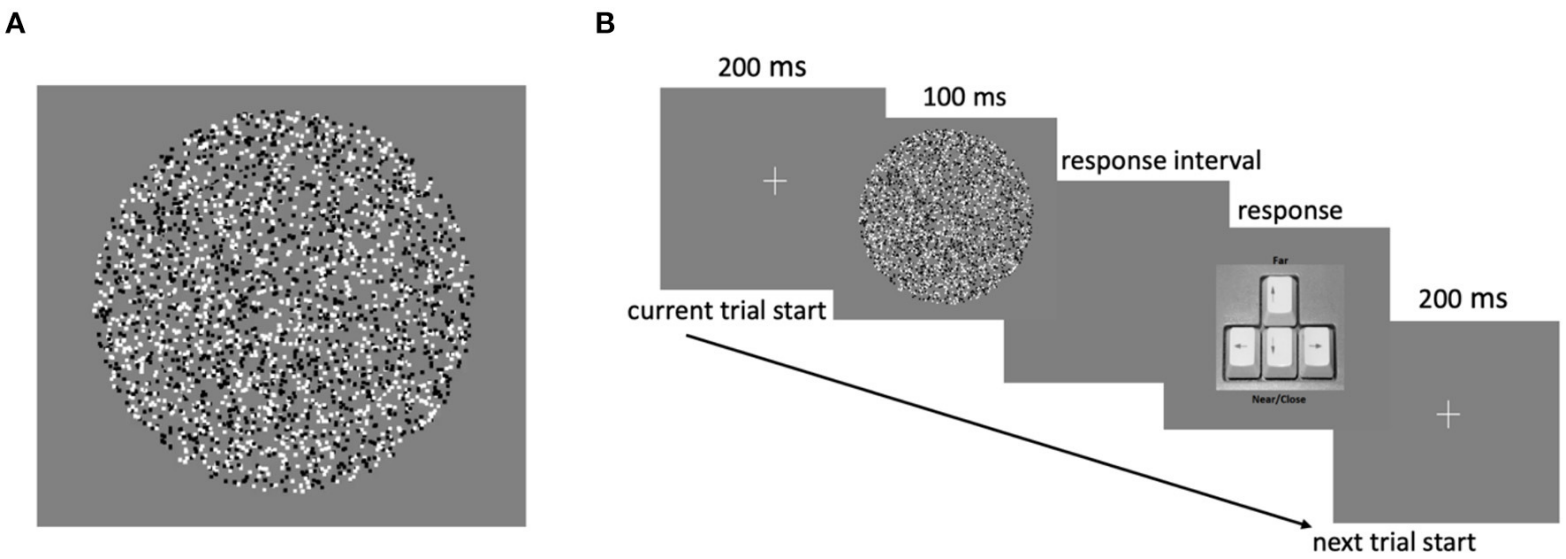
**(A)** Schematic of an RDS stimulus and **(B)** trial structure of the experimental task.

Custom-made software generated patterns of correlated RDS (i.e., dots of the same luminance were presented to each eye). The stimuli were black-and-white random dots of equal number (50% each) against a gray background (Figure 1A). The RDS stimuli were presented in a circular reference region (i.e., the surround annulus) that was always presented at 0 disparity and a test region (i.e., the center disk). Following the ranges of disparity values in a recent study that has used similar variants of RDS tasks (Asher and Hibbard, 2018) and the choice in disparity distance (ca. –/+ 5 arc min) between the test and the reference region in studies that examined perceptual learning (O'Toole and Kersten, 1992), the reference and the testing region in our study varied across six values of disparities (–/+ 5.5, –/+ 11, and –/+ 16.5 arc min). In our task, crossed (closer) and uncrossed (farther) disparities were assigned negative and positive values, respectively. There was no gap between the center disk and its surrounding. The circular reference was chosen based on findings from a previous study (Asher and Hibbard, 2018), which showed that most observers perceived forward depth with circular stimuli more than with horizontal or vertical stimuli. Furthermore, the slope of the positive function relating disparities and near-far responses is steepest using circular stimuli, which would be advantageous for assessing individual differences in disparity discrimination.

In our experiment, each trial began with a central fixation cross that appeared for 200 ms, followed by the presentation of an RDS stimulus for 100 ms. During this interval, the participants had to indicate whether the center disk appeared closer (near) or farther away (far) than the reference region. The participants reported their choices by pressing either the down or up arrow key to indicate “near” or “far” response, respectively (Figure 1B). The next trial began upon the response key press. No feedback on response accuracy was provided. Each participant completed five blocks of 90 trials each, with a total of 450 trials in the experiment. In each block, RDS stimuli with different disparities were randomly presented, with each of the six disparities presented in 15 trials.

### Apparatus

The visual RDS stimuli were presented on an ASUS 3D monitor with a spatial resolution of 1,920 × 1,080 pixels and a refresh rate of 120 Hz. The dimensions of the screen were 29.5 cm tall and 52.4 cm wide. Stereoscopic presentation of the stimuli was achieved using an NVIDIA GeForce3D Vision 2 emitter and corresponding 3D Stereo shutter glasses that the participants wore while standing ~50 cm away from the monitor. The center of the monitor was kept at the height of the eyes of the participants. Responses were recorded by using a computer keyboard. Stimuli were generated and presented using custom-made software written in C++. Laparoscopic skill training in the peg transfer task was done with a standard laparoscopic box trainer and two laparoscopic graspers. The tracking and the recording of the laparoscopic instruments (graspers) were done by using an optical tracking system (the NDI Polaris Spectra) where unique passive trackers were attached to each of the two laparoscopic graspers. These markers allowed for the two graspers to be correctly and uniquely identified with a sub-millimeter accuracy (at about 0.25 mm). Reaction times during laparoscopic training were recorded with the onset of the task until the task ended.

### Stereoacuity Test

Individual differences in stereoacuity (i.e., the smallest detectable depth difference that can be seen by binocular vision) were measured using the graded-circle test of the Titmus Fly^®^ Stereotest booklet (STEREO OPTICAL). This test comprises a graded series of 10 images that test stereopsis. At each step of the series, four circles are presented in a square, and only one of these circles has a crossed disparity, which makes it appear forward (in front) of the reference plane for individuals with normal binocular fusion.

Each participant judged which of the four circles appeared forward relative to the rest. Since variations in viewing distance can affect depth perception, the participants judged the circles while holding the test from a viewing distance of ~40 cm (i.e., converted from the viewing distance of 15 min of arc as specified by the test). The participants started with the first image in the series and continued with the other images in sequence until two successive mistakes were made. In this case, the last correct response was recorded as the measured threshold of depth discrimination. The overall performance was scored using the standard scoring chart that is provided in the manual of the test, which shows, for each correct response, the corresponding angle of stereopsis in the unit of seconds of arc.

### Laparoscopic Skill Training

**Figure 2:**
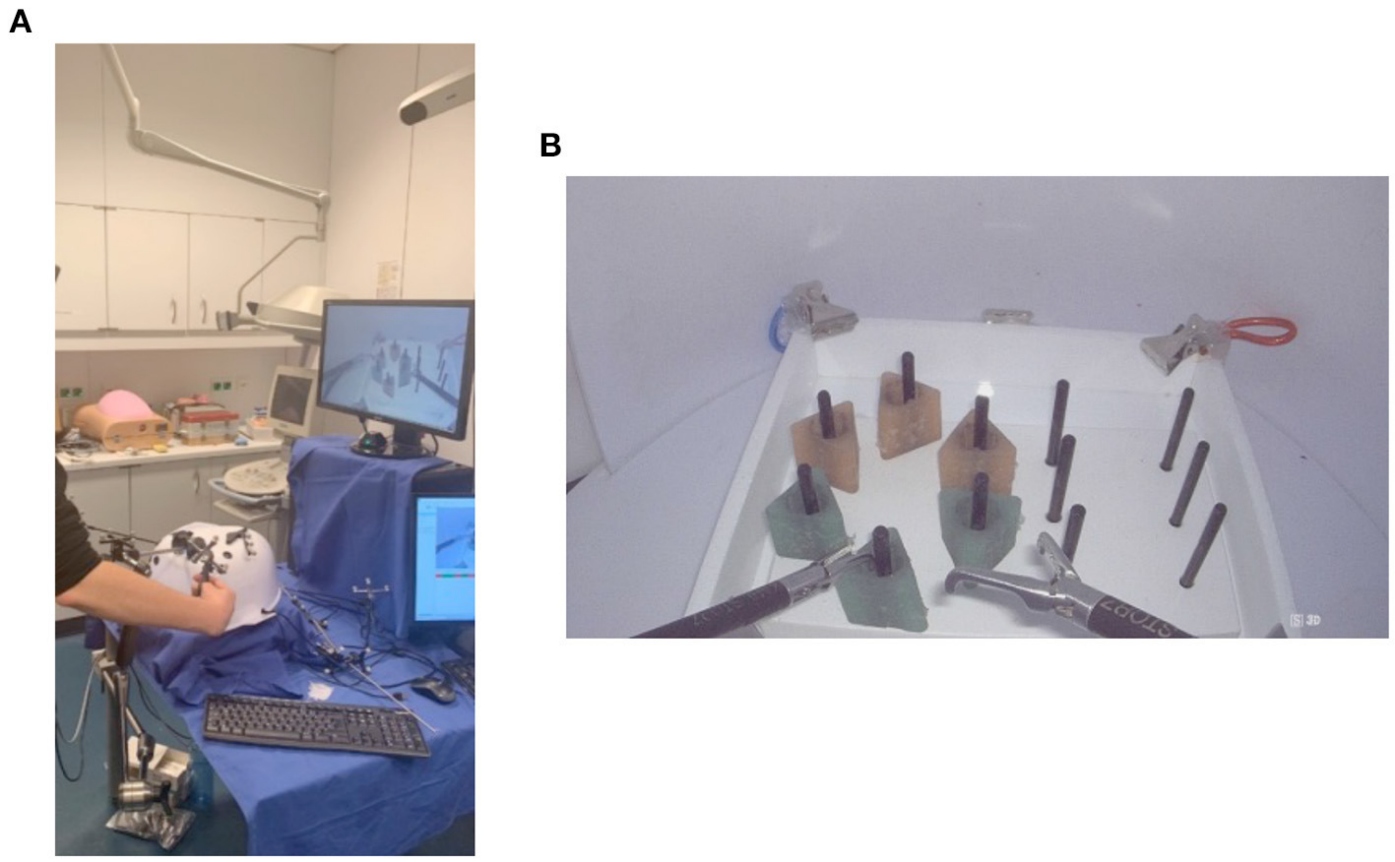
The peg transfer task performed on a box trainer during the laparoscopic skill training. **(A)** A participant stood in front of a box trainer and the 2D display while manipulating the laparoscopic graspers. **(B)** The spatial layout of the peg transfer task shown on the screen.

The participants underwent four weekly training sessions that were the very initial part of a curriculum on the Fundamentals of Laparoscopic Surgery (FLS) offered at the Department of Visceral, Thoracic and Vascular surgery of the medical faculty of Technische Universität Dresden. The FLS program is an education program with teaching tools that were developed by the Society of American Gastrointestinal and Endoscopic Surgeons (Soper and Fried, 2008; see also https://www.flsprogram.org). Worldwide, programs with similar tasks are designed and used to teach and evaluate the skills fundamental to laparoscopic surgery. Such training would be followed up with further extensive clinical training and experiences for the medical students to gain the necessary expertise of laparoscopic surgical skills. The tasks (e.g., transferring pegs, cutting a circle, stitching, and knot tying) were performed on a laparoscopic box trainer, which was placed at a viewing distance of ~40 cm from each participant. Here, we focused on assessing the performance of the peg transfer task (see Figure 2) with 2D display, because, under this situation, this task substantially requires relative depth perception. Specifically, the task of the participants was to lift each object from its peg using the laparoscopic grasper in the non-dominant hand, transfer it to the dominant hand, and then place the object on an empty peg. After all objects have been successfully transferred, the participants repeated the same procedure but in the reversed direction. Besides reconstructing 3D shapes from 2D images, performing laparoscopic skills with 2D displays in general requires eye-hand coordination and the perception of motion in depth. The peg transfer task used in the study requires tracking motion trajectories of the laparoscopic graspers in depth (Breedveld and Wentink, 2001; Bogdanova et al., 2016). The vergence of the eyes is important both for binocular eye-hand coordination (Maiello et al., 2018) and for perceiving motion in depth (Nefs and Harris, 2007). These processes are made more challenging when performing laparoscopic tasks, since visual images of the hand movements in depth are seen on a monitor on which depth cues are limited. A recent study that also used the RDS stimuli has shown that, when perceiving motion in depth, sensory control signals for vergence are very much influenced by cues of changing disparity (Giesel et al., 2019). Furthermore, even though 2D displays lack direct information about the binocular disparity, interactions between monocular motion cues and binocular disparity have been established in psychophysical data (Bradshaw and Rogers, 1996) and supported by neurobiological evidence showing that neurons in the visual cortex respond both to motion and binocular disparity [Poggio and Talbot, 1981; Maunsell and Van Essen, 1983; see also Regan et al. (1979) for an early review]. By using the RDS stimuli and random-dot kinematograms to manipulate disparity and motion, respectively, a functional brain imaging study on humans showed that activations in early visual areas (e.g., V3) respond to both types of visual information (Kohler et al., 2019). Taken together, individual differences in binocular disparity discrimination learning may be related to individual differences in learning to perform the peg-transfer task in laparoscopic settings, for which the perception of motion in depth is also important besides static depth information.

### Study Procedure

For each participant, the study began with the screening of stereopsis by assessing stereoacuity. Afterward, the participants were given a brief introduction about the experimental procedure before performing a practice block and then five blocks of the RDS-depth discrimination task. Each of the participants stood in front of the computer with the screen located at the level of eye height. In order to make sure that the stereo glasses worked properly for all the participants, before the start of the actual experimental trials, the participant had to report whether he/she could locate the letters L or R at the bottom of the screen after closing the left or right eye, respectively. After this check, the participants carried out a practice block of 18 RDS trials chosen randomly out of possible disparity values. With the completion of the practice block, the five experiment blocks of the RDS task began. The task lasted approximately 10 min. After the depth-discrimination task, the participants also underwent four separate 1.5-h sessions of laparoscopic skill training as part of the course they enrolled in. The laparoscopic training course lasted approximately 4 weeks, and the participants were trained one time every week. In the 4th week of the training, the participants also performed the RDS-depth discrimination task a second time followed by the last laparoscopic skill training.

### Overview of Statistical Methods

In order to test the effects of disparity, block, and session on depth-discrimination performance measured with the RDS task, we fitted dichotomous discrimination responses of an individual participant (“near” or “far”) across disparity with a generalized linear-mixed effect model (cf. Asher and Hibbard, 2018) that used the logit-link function in R (R Core Team, 2016). The choice of the logit-link function (Boateng and Abaye, 2019) is also motivated by signal detection theory (Green and Swets, 1966; DeCarlo, 1998) that depicts the psychophysical function of depth discrimination as logistic (cf. Gantz et al., 2007). We started by specifying a model with a maximal random effect structure (with random intercept and slopes) and stepwise simplified the model until model convergence (Barr et al., 2013). Specifically, we entered the six levels of disparity (–/+ 5.5, –/+ 11, and –/+ 16.5 arc mins), block (1 to 5), and session (1 and 2, i.e., before and after the four sessions of laparoscopic skill training) as predictors (i.e., fixed effects) along with their interactions, while the individually fitted depth discrimination response was the dependent variable. Disparity and block were modeled as continuous variables, whereas session was modeled as a categorical variable. The random effect structure of the final converged model included the participants as a random factor, with random intercepts and slopes against disparity (cf. Asher and Hibbard, 2018). We then compared this final model, which included random intercepts and slopes for the participants but fixed effects for other predictors and their interactions, with a simpler model that included only the random intercepts for the participants using the likelihood ratio test. Individual differences in disparity discrimination were assessed as variations in the estimated slopes of individual logistic disparity psychometric curves. Learning-related improvement in depth discrimination can be captured by slope changes in the disparity psychometric curve from the first to the last blocks of the experiment.

In addition, we also examined performance improvement of depth discrimination in separate generalized linear mixed-effect models of discrimination reaction time (RT) and the inverse efficiency score (computed as RT/proportion of correct responses). The performance of the participants in the peg transfer task as a function of the laparoscopic training session was analyzed using ANOVA. The disparity psychometric curves of two participants practically showed a flat (instead of the logistic) function, which reflected an absence of disparity discrimination that could either be due to a misunderstanding of the task or non-compliance to the experimental instructions. Therefore, the data of these two participants were excluded from further analyses, given that we were interested in relating individual differences in perceptual learning of depth discrimination with variations in training outcomes of laparoscopic skill acquisition. Hierarchical regression analyses were conducted to analyze these relations.

## Results

### Effects of Disparity and Perceptual Learning

We compared results from the generalized mixed-effect model with fixed effects for the predictors and their interactions, but random intercepts and slopes for the participants with the results from a simpler model that had only the random intercept for the participants as a random factor using the likelihood ratio test. The comparison showed that adding the random slopes for the disparity to the model significantly improved the model fit, ${\text{χ}}_{\left(2\right)}^{2}$ = 251.5, *p* < 0.001. Thus, we report results based on the model with random intercept and slopes for the participants against disparity and interactions between predictors. As shown in Table 1, the main effects of disparity and block were significant, whereas the effect of the session was not. The main effect of disparity indicates that the probability of far responses of the participants increased as the disparity shifted from crossed to uncrossed (signed, respectively, as negative and positive values in this study). We observe significant effects of block and its interaction with disparity. Of particular interest was the interaction between disparity × block, which indicated that the probability of far responses as a function of increasing disparity differed across blocks, reflecting steepening of the psychometric function across blocks. The lack of session effect shows that depth-discrimination performance, in general, did not differ before and after laparoscopic skill training. In the first session, when performing the RDS task, the participants were not exposed to the laparoscopic training yet. Since we were mainly interested in the relation between individual differences in disparity discrimination and laparoscopic skill learning and that session neither yielded a significant main effect nor an interaction effect with block, we thus focused on results from the second session (see Supplementary Material for individual disparity logistic psychometric curves from the first session).

**Table 1:** The generalized mixed-effects model for depth-discrimination responses with disparity, block, session, and their interactions as fixed effect predictors.

**Parameters**	**Estimate**	**SE**	**z value**	**Pr(> |z|)**
Intercept	**−0.36**	**0.11**	**−3.36**	**<0.001**
Disparity	**0.29**	**0.02**	**12.24**	**<0.001**
Block	**0.10**	**0.02**	**3.57**	**<0.001**
Session	−0.11	0.17	−0.67	0.49
Disparity × Block	**0.03**	**0.003**	**9.24**	**<0.001**
Disparity × Session	**0.13**	**0.02**	**6.33**	**<0.001**
Session × Block	0.00	0.05	0.15	0.87
Disparity × Block × Session	**−0.05**	**0.008**	**−7.37**	**<0.001**

*The participants were entered as a random factor, with random intercepts and slopes against disparity. For each model parameter, the estimated value and its standard error, along with the associated standardized z- and p-values, are reported (statistically significant coefficients are shown in boldface)*.

### Individual Differences and Block Effects of the Slopes of Disparity Psychometric Curves

**Figure 3:**
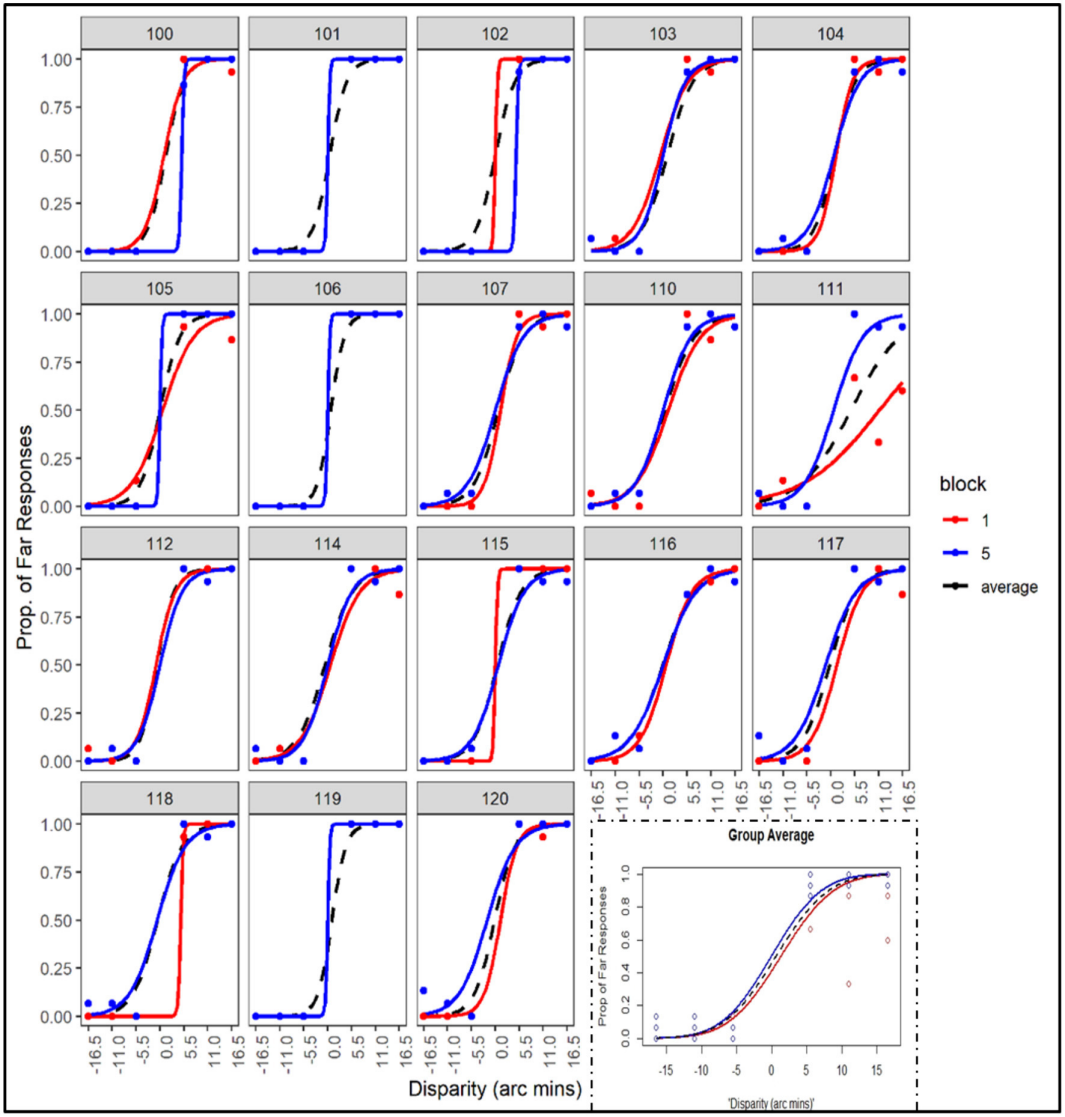
Logistic disparity psychometric curves derived by fitting the logistic function to depth-discrimination responses across disparity values (negative and positive values correspond to crossed and uncrossed disparity, respectively) for each participant. The average curves across data of all the participants are shown as the inset in the lower right-hand corner (plotted here are data from Block 1, Block 5, and the averaged data across all five blocks from Session 2). Curves from Block 1 and Block 5 are identical for three participants and are shown only in blue).

As shown in Figure 3, the slopes of the logistic psychometric function assessing depth discrimination differed between individuals and varied as a function of blocks. For visualization purposes, only the slopes from Block 1, Block 5, and the average slope across all the blocks in Session 2 are displayed for each participant (Figure 3). The slopes of the disparity psychometric curves of the participants were all positive as disparity varied from crossed (negative values) to uncrossed (positive values). Put differently, as disparity increased in magnitude in the farther direction, the probability of responding to the RDS stimuli with the “far” response increased from 0 to 1 (cf. confirmed by the significant effect of disparity in Table 1). The slopes also differed substantially between individuals, indicating notable individual differences in the ability of depth discrimination, in spite of the fact that all these participants passed the stereovision screening using the stereoacuity test (Fly^®^ Stereotest booklet). Of note, the slopes of the disparity psychometric curves also varied across learning blocks (cf. significant disparity × block interaction in Table 1). Considered as the average across individuals, the slopes of the psychometric discrimination curves numerically increased from Block 1 to Block 5 (see the inset in the right lower corner in Figure 3). Individually, the slopes of the curve in six participants in the last block (Block 5) were steeper than their individual averaged curve across five blocks and the curve based on data from the first block. Taken together, we observed substantial individual differences in slope changes of the disparity psychometric curves from Block 1 to Block 5, indicating that the extent of practice-related improvement of depth discrimination differed between the participants. A later set of analyses examined whether such individual variations may be associated with individual differences in laparoscopic learning.

### Performance Improvement in Depth Discrimination

Since it is known that speed and accuracy can trade off in many cognitive and perceptual tasks (Wickelgren, 1977) and the time to perceive depth (response latency) has been shown to reduce substantially only after a few blocks of repeated exposure to the stimuli (O'Toole and Kersten, 1992), in our task, the main performance indicator was discrimination RT, with the performance accuracy maintained at a high level across individuals by selecting relatively large disparity values (cf. O'Toole and Kersten, 1992; Asher and Hibbard, 2018). Moreover, besides accuracy, reaction time (RT) had been established as a reliable measure of depth perception in suprathreshold conditions where disparity is not very small or at the threshold (Blake et al., 1980; Horváth et al., 2018). Considered in light of drift diffusion models of perception (e.g., Petrov et al., 2011; Ratcliff et al., 2016), force-choice perceptual discrimination as implemented in our task would need more time to accumulate sufficient sensory evidence for deciding between near or far when disparity is small. Thus, we assessed performance improvement in depth discrimination across blocks by examining (i) reaction time (RT) in ms and (ii) the inverse efficiency score (IES, i.e., RT/proportion of correct responses) for each participant.

**Figure 4:**
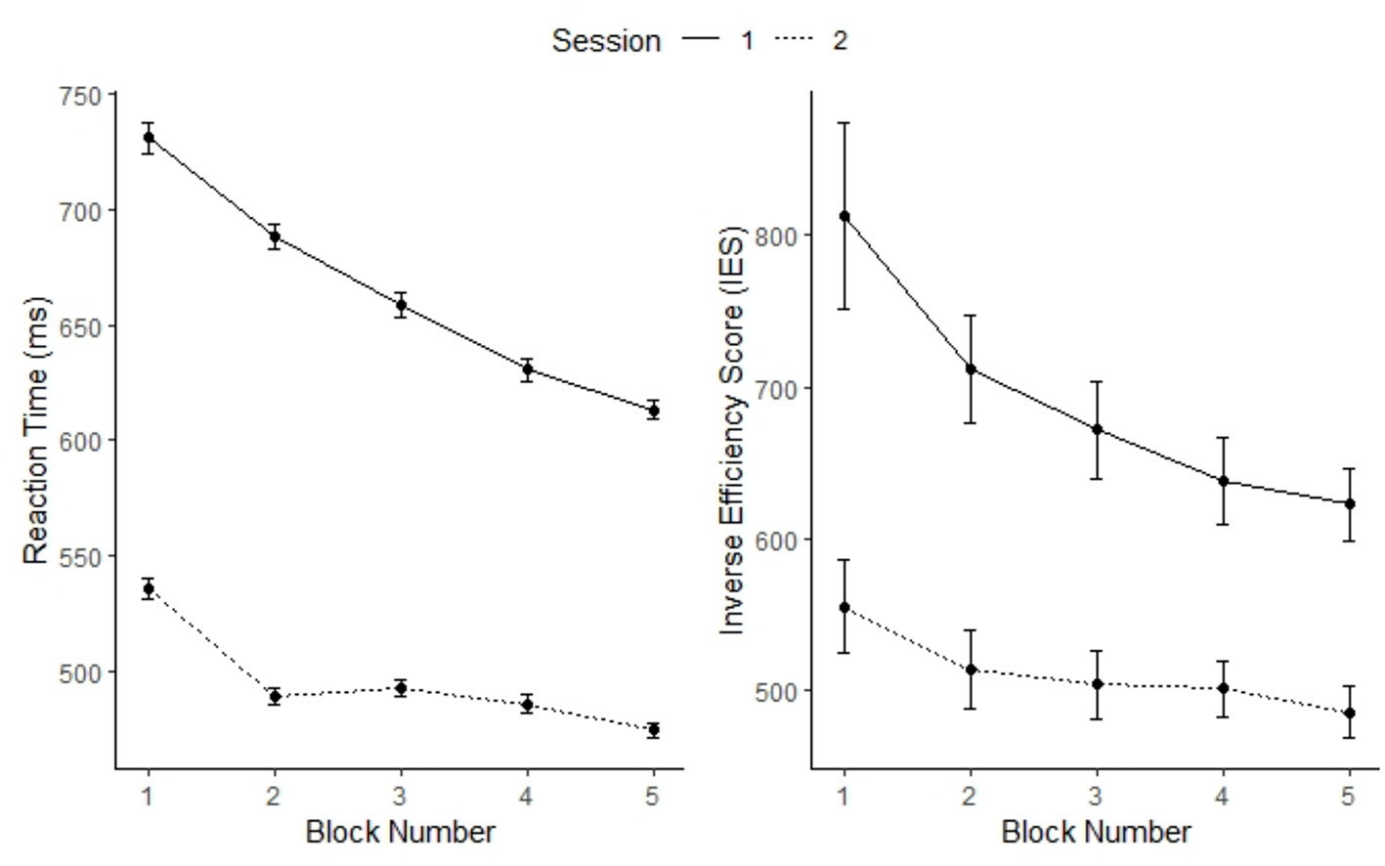
Performance outcomes of reaction time **(left)** and inverse efficiency score (IES) **(right)** as a function of session and block. Error bars represent standard errors of the means.

As expected, overall accuracy for this task was relatively high in both sessions (Session 1: M = 0.97, SD = 0.16; Session 2: M = 0.96, SD = 0.17). Data inspection also did not show evidence of speed-accuracy trade-off across the responses of the participants [*r*_(16)_ = – 0.079, *p* = 0.75]. As shown in Figure 4, there was a clear improvement from Block 1 to Block 5 in RT and IES of the participants in both sessions (for the detailed statistics regarding effects from the mixed effect models of both measures, please see Supplementary Table 1).

We further verified the measure of block-related change (Block 5 vs. Block 1) in the slopes of the logistic disparity psychometric curves by examining its relations to changes in RT or IES across blocks in Session 2. Results of the analyses showed marginal correlations between the slope and IES, *r*_(16)_ = 0.43, *p* = 0.074, and between slope and RT reduction, *r*_(16)_ = 0.45, *p* = 0.06. Together, these results show a statistic trend for a positive relation between block-related changes in the slope of the disparity psychometric curve and individual differences in depth-discrimination learning.

### Performance Measures of Laparoscopic Skill Training

Performance of the peg transfer task from the laparoscopic skill training sessions was assessed in two aspects: (i) movement speed that was measured by reaction times (RT) in seconds and (ii) the precision of hand movements that was indicated by the volume (mm^3^, i.e., cubic millimeters) of the space-spanning areas covered by the movements of the laparoscopic graspers (a larger volume reflects less movement precision). The normality assumption was checked for both variables using the Shapiro-Wilk test and by visual inspections using QQ plots. The volume data were not normally distributed (Shapiro-Wilk normality test W = 0.94376, *p* = 0.002); thus, this measure was the first log transformed before subjecting it to further statistical analyses.

**Figure 5:**
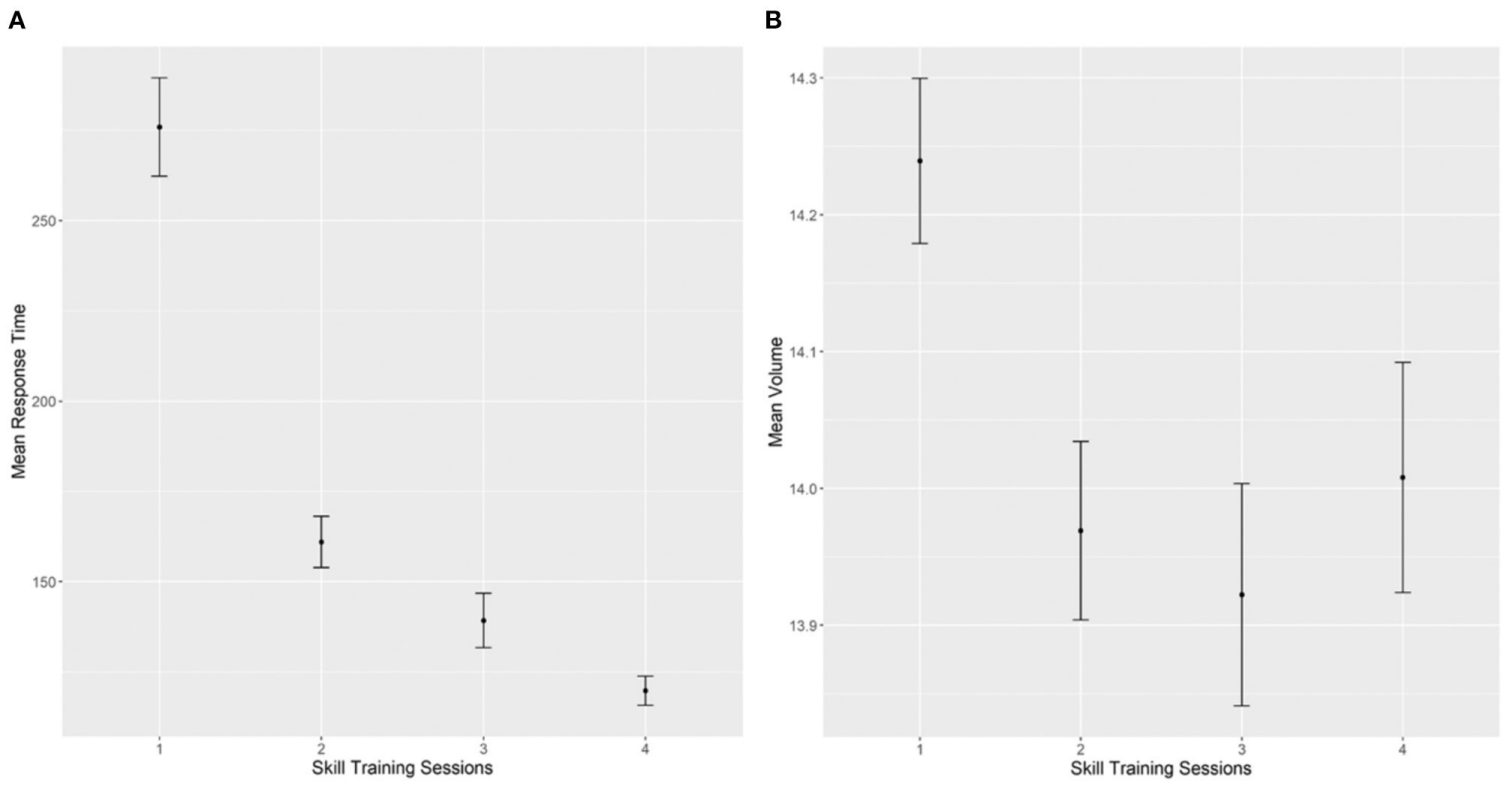
Performance outcomes of the peg transfer task across the laparoscopic skill training sessions. **(A)** Mean reaction time data (in seconds) across training sessions. **(B)** Mean log transformed volume data (mm^3^) across training sessions. Error bars represent standard errors of the means.

In terms of the speed of performance, results from the repeated measure one-way ANOVA of the RT data revealed a significant effect of the training session, *F*_(3,51)_ = 100.01, *p* < 0.001, *η*^2^ = 0.73 (see Figure 5A). This effect confirmed that, as training sessions progressed, the participants became faster (required less time) in completing the peg transfer task. Similarly, movement volume also decreased significantly across training sessions, *F*_(3,51)_ = 3.10, *p* < 0.05, *η*^2^ = 0.10 (see Figure 5B). This result confirmed that the participants were less precise in moving the laparoscopic graspers at the first training session but improved significantly as reflected in the reduced volume measure as the training sessions progressed. Due to the larger between-person variability in the measure of movement volume, the effect size of the skill training was larger for movement speed than for movement precision (*η*^2^ = 0.73 vs. *η*^2^ = 0.10, respectively).

### Relation Between Effects of Learning on Depth Discrimination and Laparoscopic Skills

In the last set of analyses, we conducted hierarchical regression analyses to investigate the relation between individual differences in perceptual learning of depth discrimination across blocks during the RDS task and performance gains across the laparoscopic training sessions. As a comparison, individual differences in stereoacuity measured by the Stereo test that is commonly used in clinical settings were also included in the regression analyses.

For each participant, we computed a *Slope Ratio* score, i.e., (Slope_*block*5_–Slope_*block*1_)/Slope_*block*1_, as a measure for individual differences in perceptual learning of depth discrimination in the RDS task. An advantage of this measure is that it controls for individual differences in disparity at the baseline. The performance gains after laparoscopic skill training (i.e., differences between training session 1 and session 4) were computed for RT and an *efficiency* score that combined RT and volume into one common measure [i.e., reaction time/log (volume)]. The stereoacuity score assessed by the standard graded-circle StereoTest was measured in seconds of arc (i.e., arc sec) and was log transformed as suggested by previous studies (Adams et al., 2009; Zhao and Wu, 2019) and labeled as *LogArcsecs*. Since the measurement scales of these variables differed widely, all variables were first standardized to the comparable scale of z-scores before submitting them to the regression analyses. Tables 2, 3 below show results from two sets of hierarchical regression analyses that related individual differences in perceptual learning of depth discrimination and stereoacuity with between-person variations in performance gains of RT (Table 2) and the combined *efficiency score* (Table 3).

**Table 2:** Results of a pair of hierarchical regression models predicting performance gains of RT during skill training.

**Predictor**	***b***	***Beta***	***sr*** ^**2**^	***r***	***Fit***	**Difference**
**Regression 1: depth discrimination learning (Slope Ratio) entered before stereoacuity (LogArcSecs)**						
(Intercept)	2.11^**^					
Slope Ratio	0.48^*^	0.57	0.32	0.57^*^		
					***R^2^*** = 0.322^*^	
(Intercept)	3.38^*^					
Slope Ratio	0.57^**^	0.68	0.37	0.57^*^		
LogArcSecs	−0.85	−0.25	0.05	0.04		
					***R^2^*** = 0.374^*^	Δ*R^2^* = 0.052
**Regression 2: stereoacuity (LogArcSecs) entered before depth discrimination learning (Slope ratio)**						
(Intercept)	1.90					
LogArcSecs	0.14	0.04	0.00	0.04		
					*R^2^* = 0.002	
(Intercept)	3.38^*^					
LogArcSecs	−0.85	−0.25	0.05	0.04		
Slope Ratio	0.57^**^	0.68	0.37	0.57^*^		
					***R^2^*** = 0.374^*^	**Δ*****R^2^*** = 0.373^**^

*Estimated unstandardized (b) and standardized (beta) regression weights, correlation (r), semi-partial correlation coefficients (sr^2^), fit (R^2^), and incremental fit (difference) are shown here (statistically significant effects are shown in boldface*,

* *p < 0.05*,

** *p < 0.01)*.

**Table 3:** Results of a pair of hierarchical regression models predicting gains of performance efficiency during skill training.

**Predictor**	***b***	***beta***	***sr^2^***	***R***	***Fit***	**Difference**
**Regression 1: depth discrimination learning (Slope ratio) entered before stereoacuity (LogArcSecs)**						
(Intercept)	2.13^**^					
Slope Ratio	0.49^*^	0.58	0.34	0.58^*^		
					***R^2^*** =0.336^*^	
(Intercept)	3.32^*^					
Slope Ratio	0.58^**^	0.68	0.38	0.58^*^		
LogArcSecs	−0.80	−0.24	0.05	0.06		
					***R^2^*** =0.382^*^	Δ*R^2^* = 0.045
**Regression 2: stereoacuity (LogArcSecs) entered before depth discrimination learning (Slope ratio)**						
(Intercept)	1.82					
LogArcSecs	0.21	0.06	0.00	0.06		
					*R^2^* = 0.004	
(Intercept)	3.32^*^					
LogArcSecs	−0.80	−0.24	0.05	0.06		
Slope Ratio	0.58^**^	0.68	0.38	0.58^*^		
					***R^2^*** =0.382^*^	**Δ*****R^2^*** =0.378^**^

*Estimated unstandardized (b) and standardized (beta) regression weights, correlation (r), semi-partial correlation coefficients (sr^2^), fit (R^2^), and incremental fit (difference) are shown here (statistically significant effects are shown in boldface*,

* *indicates p < 0.05*.

** *indicates p < 0.01.)*.

In the first set of analyses, individual differences in depth-discrimination learning (indicated by the variable, *slope ratio*) were either entered before or after stereoacuity (the *LogArcsecs* variable) as an independent variable to account for individual differences in gaining speed (faster RT) when performing the peg transfer task after skill training. The pair of analyses was conducted to discern whether depth-discrimination learning is a more sensitive predictor for individual differences in laparoscopic skill acquisition than the standard measure of stereoacuity. Specifically, the results showed that *slope ratio*, when entered at the first step, significantly accounted for 32.2% of variance in individual differences in RT gains, *F*_(1,16)_ = 7.61, *p* < 0.05. Adding individual differences in stereoacuity (the LogArcSecs variable) at the second step of the hierarchical regression increased the total amount of explained variance to 37.4%, *F*_(2,15)_ = 4.48, *p* < 0.05. However, the increase of an additional 5.2% of explained variance was not significant. These results indicated that stereoacuity was not a unique predictor for individual differences in performance gain of RT as a function of skill training. These results were further confirmed by findings from a second regression in which predictors were entered in the reversed order. When stereoacuity (*LogArcSecs*) was entered as a predictor at the first step, it did not significantly account for laparoscopic performance gains in RT, *F*_(1,16)_ = 0.02, *p* = 0.86 (only 0.2% of variance explained). However, adding *slope ratio* to the model at the second stage, which controls for individual differences in stereoacuity, still accounted for an additional 37.3% of individual variations in performance gains of RT, which is significant (*p* < 0.05). Taken together, results from this pair of regression analyses showed that, whereas individual differences in depth-discrimination learning were associated with 37.3% of variances in individual differences of performance gains during laparoscopic skill training, stereoacuity did not account for a significant amount of variance in training gains.

The second set of regression analyses was done to account for between-person variations in performance improvements with respect to the *efficiency score* across the skill training sessions. The analyses were conducted in the same manner as the analyses reported above. In general, the patterns of results were very similar to the results regarding performance gains of RT. Specifically, the results of the second pair of regressions showed that *slope ratio* when entered at the first step significantly accounted for 33.6% of variance in improvements of the *efficiency score, F*_(1,16)_ = 8.11, *p* < 0.05. Adding individual differences in stereoacuity (the *LogArcSecs* variable) at the second step increased the total amount of explained variance to 38.2%, *F*_(2,15)_ = 4.48, *p* < 0.05. However, the increase of an additional 4.6% of explained variance was not significant. These results indicated that stereoacuity was not a unique predictor for individual differences in performance gains of the *efficiency score*. These results were further confirmed by findings from a second regression that entered the predictors in the reversed order. When stereoacuity (*LogArcSecs*) was entered as a predictor at the first step, it did not significantly account for laparoscopic performance gains of the *efficiency score, F*_(1,16)_ = 0.58, *p* = 0.81, and accounted only for 0.4% of the variance. However, adding *slope ratio* to the model at the second stage, which controls for individual differences in stereoacuity, is still significantly accounted for an additional 37.8% (*p* < 0.05) of individual variations in performance gains of the *efficiency score*. Together, the results from both pairs of regression analyses clearly showed evidence that, whereas individual differences in depth discrimination accounted for substantial amounts of variances in two measures of performance gains during laparoscopic skill training, the relation between stereoacuity and performance gains after laparoscopic skill training was weak and could not account for much of the individual variations.

## Discussion

Considering the greater demand for the depth perception ability of the surgeons for performing laparoscopic surgery relative to traditional open surgery (Breedveld et al., 1999; Bogdanova et al., 2016) and the plasticity of depth perception (Chowdhury and DeAngelis, 2008; Başgöze et al., 2018), this study investigated whether individual differences in depth-perceptual learning may be related to variations in training-related laparoscopic skill improvements in medical students who were still naïve of such minimally invasive surgical procedures. Besides screening stereoacuity with the standard Stereotest (cf. Sakata et al., 2017a,b) that is commonly used in clinical settings, we assessed individual differences in the ability of binocular depth discrimination and its learning-related improvement by using computer-generated RDS stimuli. Results of the study showed that individuals differ substantially in their ability of depth discrimination that is reflected in the slopes of the individual disparity psychometric curves. Furthermore, a significant amount of between-person variations in the effect of learning on depth discrimination, as reflected in slope changes from the first to the last experimental blocks, was also evident. Of particular relevance is the finding that individual differences, in such perceptual learning and performance gains of laparoscopic skill training, are correlated as hypothesized. Individual differences in learning-related changes in the slopes of the disparity psychometric curves were associated with over 37% of variance in variations of training-related performance gains when performing the peg-transfer task. This result was observed both with respect to performance speed and an efficiency score that combines movement speed and precision. Moreover, unlike contour stereotests, which evaluate two horizontal disparate stimuli *via* a vectographic technique in which the observer views a stereoscopic print *via* polarized 3D glasses (Fricke and Siderov, 1997), the assessment of psychometric curves of the participants *via* the RDS paradigm could account for variations in laparoscopic skill learning, whereas the basic measure of stereoacuity showed inconsistent results (Fawcett and Birch, 2003; Sakata et al., 2017a,b).

On the one hand, technological advances on refining the 3D display mode of laparoscopy *via* double lenses or other methods could aid binocular vision and reduce errors (see Sinha et al., 2017; Gabrielli et al., 2020). When considering the time of operation as the primary outcome, systems that provide 3D vision have been shown to be advantageous in some clinical settings (e.g., Itatani et al., 2019). Results from experimental settings (e.g., using a standardized laparoscopic box trainer) have thus far also shown that, when trained with 3D display, learning speed and precision can be improved, at least in some studies (e.g., Beattie et al., 2020; Kunert et al., 2020). However, the transfer of performance trained under 3D display to actual operative situations and long-term clinical outcomes is still equivocal (Yim et al., 2017; Beattie et al., 2020). On the other hand, even though 3D display could make depth perception in laparoscopic surgical settings less demanding, normal stereopsis is a prerequisite. Moreover, studies have shown that about 2–14% of evaluated surgeons had reduced stereopsis (Biddle et al., 2014) or did not have measurable stereopsis (Fergo et al., 2016). In the general population, the average prevalence of “stereoblindness,” i.e., the total absence of stereoscopic vision is estimated to be around 7% but varies greatly in the range from 1 to 30%; furthermore, the prevalence rate increases with age during adulthood (Fergo et al., 2016; Chopin et al., 2019). Specifically, evidence from a cross-sectional study with 300 medical doctors (25–71 years) showed that older age (being in the age group > 50 years) was significantly associated with problems of stereovision (Fergo et al., 2016). Another study with more than 100 regular (non-physician) participants (age between 15 and 79 years), who were tested with the RDS task showed that stereoacuity decreases with age, with the stereo threshold increasing from about 30 arcsec in adolescence and young adulthood to about 140 arcsec in the 8th decade in life on average (Zaroff et al., 2003). Furthermore, a study showed that, for individuals with suboptimal stereoscopic vision, performances of fine motor tasks under laparoscopy were worse with 3D than with 2D display (Bloch et al., 2015). Thus, independent of whether laparoscopic tasks can be performed with or without technically augmented 3D displays, perceptual training that can potentially improve depth perception could be a helpful psychophysical intervention approach to enhance depth-discrimination ability in medical students and surgeons.

In line with previous research on perceptual learning in general (Dosher and Lu, 1999; Gilbert et al., 2001; Fahle, 2005) and the plasticity of depth perception (Gantz et al., 2007; Chowdhury and DeAngelis, 2008; Başgöze et al., 2018), at the group level, we observed an increase in the slope of the disparity psychometric curve across experimental blocks that took only about 10 min of repeated exposures to the RDS stimuli. In individuals with abnormal binocular vision (e.g., strabismus, anisometropic, or amblyopia), extensive perceptual learning has been shown to help recover stereopsis (Ding and Levi, 2011; Xi et al., 2014). Going beyond these prior findings, this study shows that individual differences in the effects of learning on disparity discrimination are associated with performance gains from laparoscopic skill training.

Previously, it has been shown in rhesus monkeys that perceptual learning of depth discrimination across experimental blocks increases the involvement of perceptual decision processes subserved by parietal and frontal brain regions, instead of primary reliance on visual sensory processes implicated by the visual cortex and the extrastriate visual cortex (Chowdhury and DeAngelis, 2008). Repeated exposures to the RDS stimuli have also been suggested to reduce perceptual noise (Gantz et al., 2007). Juxtaposing these earlier findings with the results of this study, it could be supposed that beneficial perceptual learning effects across blocks of the RDS task might be related to reduced processing noise and fine-tuned perceptual priors (Knill, 2007) that strengthen perceptual decision. The involvement of higher-order cognitive processes during depth perception is also required when acquiring and performing laparoscopic skills. Taken together, these findings hint at the possibility of using perceptual learning paradigms as potential intervention approaches to enhancing the learning effects of laparoscopic skill training.

However, the current study is limited in assessing depth perception with only one specific visual task and offers only correlational evidence. Future research that more systematically varies different sensory cues (e.g., monocular and binocular) and sensorimotor demands (e.g., visual-motor coordination) is necessary to systematically investigate potential mechanistic relations between depth perceptual learning and laparoscopic skill acquisition. It should also be kept in mind that, other than individual differences in binocular depth discrimination, general learning effects (e.g., increased cognitive and motor speeds) may have also contributed to the observed relation between depth perception learning and performance gain during laparoscopic skill training. The effects observed here still need to be further verified in future studies using finer disparity values as well as more extensive depth discrimination learning. Whereas, we have demonstrated that the sensitivity of binocular disparity discrimination can be modified after a relative short learning across a few experimental blocks (cf. Wallach and Karsh, 1963; O'Toole and Kersten, 1992), longer training protocols involving several thousands of learning trials may be necessary for establishing more general transferable effects (cf. Gantz and Bedell, 2010). Furthermore, only binocular disparity discrimination is examined in this study; future studies need to consider using dynamic RDS tasks to investigate individual differences in the ability to perceive motion in depth (e.g., Nefs et al., 2010), as well as other monocular cues for depth and their interactions with binocular information. Although the measured stereoacuity in our sample reflected the distribution observed in college populations (Deepa et al., 2019), three of our participants showed reduced stereoacuity as measured by the Titmus Fly^®^ Stereotest. Future studies on relations between disparity discrimination and laparoscopic skills with larger samples should consider more stringent screening for inclusion.

In order to help translate these findings into medical education of laparoscopic skill training or clinical practices, future applied research would benefit from basic neuroscience research that combines similar RDS paradigms in humans with brain imaging (functional magnetic imaging or functional near-infrared spectroscopy) and computational modeling (e.g., Bitzer et al., 2014) to more closely investigate the influences of perceptual learning of depth discrimination on neuronal noise tuning (e.g., Li et al., 2006; Li and Rieckmann, 2014) and adjustment of perceptual priors (e.g., Knill, 2007) and how these may affect functional redistributions in brain circuitries involved in the perceptual decision and sensory processing during depth perception. In terms of refining the RDS paradigm as a psychophysical intervention approach, studies that more systematically explore different stimulus features (e.g., Asher and Hibbard, 2018), as well as the amount of exposures and size of disparity in individuals of different ages and levels of laparoscopic skills, would be helpful. Applied research that explores methods and platforms for integrating the RDS paradigm into virtual-reality-based simulators for laparoscopic skill training (cf. Lucas et al., 2008) could also be useful.

## Conclusion

Sensitive depth perception is an important ability required for performing laparoscopic surgery. Individuals differ not only in stereoacuity but also in their potentials to benefit from learning to improve depth discrimination. In line with previous research, we observed that short-term exposures to RDS with varying degrees of disparity can enhance the depth discrimination, presumably through enhanced perceptual attention and decision processes and their underlying brain mechanisms. Furthermore, previous results were extended in the new finding, showing that individual differences in depth discrimination learning are correlated with performance gains from laparoscopic skill training in novice. Other than augmenting medical students and surgeons with advanced visual displays to reduce high demands for depth perception during laparoscopic skill training or in clinical situations, psychophysical intervention paradigms might be a potentially complementary approach. To this end, more research is needed to better understand the mechanisms underlying the here observed association between the effects of learning on depth discrimination and laparoscopic skills.

## Data Availability Statement

The anonymized raw data supporting the conclusions of this article will be made available by the authors upon request, without undue reservation.

## Ethics Statement

The studies involving human participants were reviewed and approved by the ethical committee of Technische Universität Dresden (No. EK285072017). The participants provided their written informed consent prior to participating in this study.

## Author Contributions

AH, SB, FB, SS, and S-CL conceptualized and designed the study. The software for the RDS task and for quantitative assessments of the peg transfer task were developed by SB. Data collection of the RDS task was done by AH, whereas FB, IF, FO, MD, and JW were involved in course development and/or assessing medical students' performance during laparoscopic skill training sessions. AH and S-CL conducted data analyses and wrote the first version of manuscript. SB, FB, and SS contributed to later versions of the manuscript. All authors approved the publication of this manuscript.

## Funding

This study was funded by the Excellence Strategy of the German Research Foundation (Deutsche Forschungsgemeinschaft) DFG EXC 2050/1 Project ID 390696704 – Cluster of Excellence Centre for Tactile Internet with Human-in-the-Loop.

## Conflict of Interest

The authors declare that the research was conducted in the absence of any commercial or financial relationships that could be construed as a potential conflict of interest.

## Publisher's Note

All claims expressed in this article are solely those of the authors and do not necessarily represent those of their affiliated organizations, or those of the publisher, the editors and the reviewers. Any product that may be evaluated in this article, or claim that may be made by its manufacturer, is not guaranteed or endorsed by the publisher.
