# Supplementary material for: Associations Between Binocular Depth Perception and Performance Gains in Laparoscopic Skill Acquisition
Source: Front Hum Neurosci. 2021 Oct 5;15:675700. doi: 10.3389/fnhum.2021.675700 (PMC8524002; doi:10.3389/fnhum.2021.675700)
Supplement: Supplementary file 1 [file Data_Sheet_1.pdf]

Supplementary Materials

Hatzipanayioti et al. *Associations between Binocular Depth Perception and Performance Gains in Laparoscopic Skill Acquisition*.

Figure S1

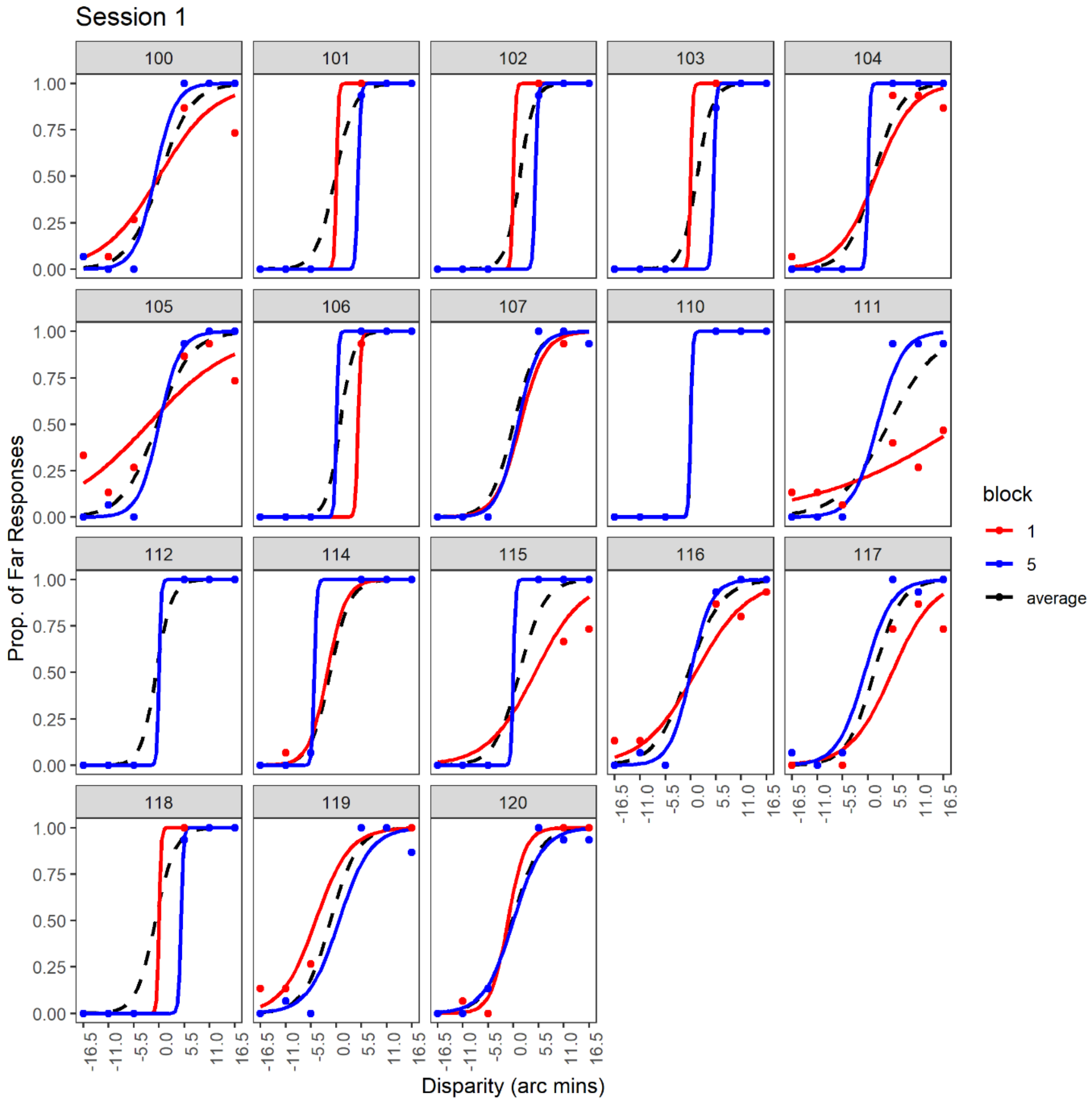

Figure S1. Performance of the RDS task in Session 1. Session 1 was carried out before the participants started with the laparoscopic skill training. Logistic disparity psychometric curves derived by fitting the logistic function to depth discrimination responses across disparity values (negative and positive values correspond to crossed and uncrossed disparity, respectively) for each participant. Plotted here are data from Block 1, Block 5, and the averaged data across all five blocks (curves of Block 1 and Block 5 are identical for two participants and are shown only in blue).

Performance in depth discrimination. In Table S5 only results from the full models with random intercepts for participants along with the interactions of the fixed predictors are shown, since including interactions improved model fit for both Inverse Efficiency Score (χ^2 (4) = 14.61, $p < .001$) and response latency (χ^2 (4) = 98.00, $p < .001$) respectively. The analysis revealed that Block and Session significantly predicted both Inverse Efficiency Score and reaction time. Notably, there was a significant interaction between Block x Session for both Inverse Efficiency Score and reaction time, which indicated that performance differed across blocks and sessions in both measures.

Table S1. Generalized mixed-effects model for depth discrimination responses in Inverse Efficiency Score (IES) and Response Latency with Block, Session and their interactions as fixed predictors. Participants were entered as a random factor with random intercepts. For each model parameters, the estimated value and its standard error along with the associated standardized *z*-, t- and *p*-values are reported (statistically significant coefficients are shown in boldface).

	Inverse Efficiency Score				Reaction Time			
**Parameters**	**Estimate**	**Std. Error**	**t value**	**Pr(> |z|)**	**Estimate**	**Std. Error**	**t value**	**Pr(> |z|)**
Intercept	827.09	31.55	26.20	**<.001**	754.85	24.373	30.96	**<.001**
Block	-45.26	5.43	-8.33	**<.001**	-30.03	1.22	-24.57	**<.001**
Session	-268.94	25.46	-10.56	**<.001**	-219.78	5.72	-38.40	**<.001**
Session x Block	30.03	7.67	3.91	**<.001**	17.02	1.71	9.92	**<.001**
